# Interdisciplinary Approaches for Integrating Materials Science and Dentistry

**DOI:** 10.3390/bioengineering10030344

**Published:** 2023-03-09

**Authors:** Galvin Sim Siang Lin, Carlos A. Jurado, Kelvin I. Afrashtehfar

**Affiliations:** 1 Department of Dental Materials, Faculty of Dentistry, Asian Institute of Medicine, Science and Technology (AIMST) University, Bedong 08100, Kedah, Malaysia; 2 Department of Prosthodontics, The University of Iowa College of Dentistry and Dental Clinics, Iowa City, IA 52242, USA; 3 Evidence-Based Practice Unit, Clinical Sciences Department, College of Dentistry, Ajman University, Ajman City P.O. Box 346, United Arab Emirates; 4 Department of Reconstructive Dentistry and Gerodontology, School of Dental Medicine, University of Bern, 3010 Bern, Switzerland

## 1. Background: Interdisciplinary Teaching for Better Dental Care Materials Selection

Dental materials science is a core course in most undergraduate dental curricula. It covers materials science and dentistry with traditional and novel concepts. However, such a course is typically taught as a standalone course with no or little integration with other dental disciplines. This has raised concerns, as complex patient oral care demands a comprehensive interdisciplinary approach. As a result, interdisciplinary approaches in teaching and learning dental materials science courses are particularly crucial in the swiftly evolving world to produce dental graduates who can integrate their knowledge of basic materials science and translate it into their clinical practice, especially when selecting suitable materials for high-quality treatment outcomes. Therefore, this editorial aims to provide an overview of the current state and interdisciplinary approach in delivering dental materials science courses. It also highlights the challenges and opportunities for future interdisciplinary-based dental education.

Dental schools around the world are implementing innovative approaches to incorporate interdisciplinary education into their dental materials science courses. For example, some dental schools, such as Ajman University, are adopting a problem-based learning approach. There, students are presented with complex case scenarios that require integrating knowledge from multiple disciplines to develop a comprehensive treatment plan. Other schools are incorporating hands-on workshops and laboratory experiences to help students to better understand dental materials’ properties and behaviors. Additionally, some schools are partnering with engineering or materials science departments to offer joint courses that provide a more in-depth understanding of materials science concepts and their application in dentistry. These examples illustrate the potential for interdisciplinary approaches in dental education to produce graduates that are better equipped to provide high-quality patient care.

## 2. Current State of Materials Science in Dentistry

Most dental schools divide their undergraduate dental curricula into preclinical and clinical phases. Dental materials science is a core course that covers the properties of the materials used for patient care [1]. It is often introduced either as a standalone course or a series of individual modules that spread across other dental courses or into the preclinical phase of most undergraduate dental curricula. Dental materials science courses address the fundamental properties (i.e., mechanical, physical, chemical, and biological properties), handling, requirements, chemical compositions, setting reactions, characteristics, and applications of various dental materials in both clinical and laboratory settings. Dental materials science courses typically start with a topic of introduction to dental materials and further focus on clinical-based and laboratory-based dental materials. Examples of clinical-based dental materials are impression materials and dental restorative materials, as well as dental abrasives and polishing materials. Meanwhile, laboratory-based dental materials include dental waxes, dental ceramic materials, denture-based polymer materials, metal and alloys, and dental gypsum products. Moreover, cutting-edge technology and newly developed biomaterials fabricated with subtractive and additive technology were incorporated into the syllabus [2,3,4,5,6].

On the other hand, some dental schools divided the course into various discipline-based dental materials, such as those used in operative dentistry, prosthodontics, paediatric dentistry, orthodontics, or periodontics. As dental students should be equipped with the knowledge and skills to select, handle and apply dental materials in both clinical and laboratory settings, a superficial comprehension of the properties and characteristics of dental materials may be insufficient [2]. Unsurprisingly, some dental schools still need to implement a discipline-based approach to teaching dentistry, in which the curriculum is divided into various dental disciplines, including dental materials science. The discipline-based or subject-based curriculum is also known as a traditional curriculum, which refers to a wide range of dental specialties or disciplines that are bound together in the curriculum contents [7,8]. Although discipline-based curricula frequently focus on teaching students precise, up-to-date knowledge and skills in a specific course, students may still find it challenging to grasp the course’s integration of multidisciplinary knowledge, due to the fragmented discipline-based curricula [4]. The lack of relevance in translating the knowledge of dental materials science to clinical or laboratory workplaces has also rendered students to occasionally perceive dental materials science only as dull and a requirement to fulfil [9,10,11,12].

Discipline-based curricula prevent dental students from understanding the broader context of what they have learned in a specific discipline. For instance, in a dental materials science discipline-based curriculum, all topics were taught in isolation without participating in other dental disciplines, particularly conservative dentistry, and prosthodontics. Students may not know how dental materials interact with other disciplines or how different dental materials may impact the fabrication of dental prostheses. Furthermore, students may need to be made aware of how to use different dental materials in different aspects of dental treatment to provide a preventative or invasive oral health service. Hence, dental educators must explore interdisciplinary approaches in the teaching and delivery of dental materials science.

## 3. Interdisciplinary Approaches in Dentistry and Materials Science

The complexity and breadth of the educational context using an interdisciplinary approach in dental curricula reflect the growing need for dental graduates with the knowledge and skills to treat patients in multidisciplinary settings [13,14,15,16,17]. However, without initially understanding interdisciplinary dental practice, it would be challenging to debate interdisciplinary dental education. Interdisciplinary is a means of addressing a complex issue that cannot be solved through one specific field or profession. Integrating ideas and guiding principles from several disciplines to create an analytical framework that is broad and ideally cohesive to provide a deeper understanding of the topic is characteristic of interdisciplinary education [18]. In interdisciplinary dental education, the fundamental and clinical science curricula are integrated to provide clinically relevant basic science education and scientifically grounded clinical care training [13]. To ensure competence in clinical decision-making, future dental care providers must have the ability to integrate scientific knowledge from multiple dental disciplines. For example, students utilise their knowledge of the basic properties of dental biomaterials in selecting appropriate materials for treating dental caries in conservative dentistry.

The initial stage is to combine the “siloed” standalone dental materials science courses into other dental disciplines through vertical and horizontal integration. In a multi-layered curriculum, integration may be supported in several ways, including horizontal and vertical integration. When knowledge, skills, and attitudes from different disciplines are combined and learned at roughly the same time, it is referred to as “horizontal integration.” Meanwhile, the term “vertical integration” describes the blending of knowledge, skills, and personal qualities learned during the preclinical phase and advancement to the clinical phase [19]. Faculty members from other disciplines must also collaborate to review and map out the existing course content, learning objectives, pedagogical strategies, assessments, learning opportunities, and course scheduling, which is also known as “curriculum mapping” [20]. Students’ opinions on the existing curriculum should be taken into consideration. At this point, curriculum mapping aims to determine which dental materials science topics may be broken down into smaller parts and parked under other dental specialties, with less focus on compartmentalised teaching and assessment methods.

In horizontal integration, dental materials science courses can be divided based on their application in different dental specialities. For example, dental composite resin and dental cement topics can be placed under conservative dentistry disciplines. In contrast, topics such as dental ceramic materials and metal and alloys for prostheses can be integrated into the prosthodontic discipline. Moreover, advanced material and biomedical engineering knowledge can be incorporated into the topic contents. Additionally, topics such as dental stem cells, tissue engineering, regenerative dentistry, and nanotechnologies could be integrated into the undergraduate dental curriculum and linked to the basic medical sciences discipline, such as biochemistry and biomedical science. This approach would enable students to connect the fundamental principles of material sciences and bioengineering with the concepts in dentistry.

On the other hand, vertical integration can happen by categorising dental materials science courses across the undergraduate curriculum from the preclinical to clinical phase. Dental materials in prosthodontics can be taken as an example. In the preclinical phase, students are exposed to the properties, and compositions of acrylic resin and cobalt chrome used in removable prostheses. When students have fulfilled the requirements to start the clinical phase, they are taught to justify the selection of these materials in managing specific cases based on the advantages, disadvantages, indications, and contraindications of those materials. Furthermore, topics such as emerging biomaterials and advanced technologies could be introduced during the clinical years of the undergraduate dental curriculum, which would aid in building their basic knowledge in dental materials science. Thus, it is reasonable to state that the horizontal and vertical integration of materials science and various fields of dentistry will enable students to develop critical thinking skills and achieve the desired competency in delivering patient care.

The disciplines taught by faculty members in prior years may not always be known in traditional discipline-based curricula. This makes it challenging for teachers to accurately identify what students have learned and guide them in making conceptual connections between materials science and dentistry. Therefore, in response to the contemporary advancement in dental education, dental schools must implement an interdisciplinary approach in teaching dental materials science courses, as future dental graduates must be able to evaluate and critically apply various materials in real-world clinical scenarios. Such an interdisciplinary approach supports a constructivist paradigm, which enables the creation of new knowledge and a deeper comprehension of introductory concepts among students [21]. In other words, when students are exposed to novel biomaterials, they can relate and apply its usage to what they have learned in other dental disciplines, such as conservative dentistry, prosthodontics, periodontics, or paediatric dentistry.

## 4. Challenges and Opportunities for Future Research

Collaboration between educators is one of the main obstacles to achieving genuine interdisciplinary learning in dental educational settings. It may be challenging, but not impossible. The effectiveness of interdisciplinary teaching and learning is increased when experts from different disciplines collaborate towards a shared learning goal and assist students in making connections between different fields of dentistry. Dental schools should consider including adjunct faculties from different disciplines to deliver dental materials science content. Teaching staff with basic sciences backgrounds such as material science, chemistry, chemical engineering, or bioengineering will ensure their involvement in the teaching and learning of the fundamental principles, properties, compositions, and chemical reactions of dental materials, as well as advanced technologies such as nanotechnology and regenerative materials in dentistry.

Conversely, teaching staff with a basic dental degree can help to guide students to translate materials science knowledge into clinical dentistry. There are several ways to support collaborative teaching, including the following: one teach, one support; parallel teaching; alternative teaching; station teaching; and team teaching [22]. Such collaborative teaching improves the quality of education, as different teachers with various educational backgrounds (materials science, chemistry, chemical engineering, bioengineering, dental science, among others) approach the same topic from different perspectives. Nonetheless, it should also be highlighted that no single staff member with a specific educational background is recommended to cover all aspects of teaching and learning in dental materials science.

In addition, various innovative pedagogical strategies should be implemented to promote active engagement and allow students to connect the content of materials science with dentistry [23]. Undeniably, the implementation of an integrated dental materials science curriculum is incredibly challenging, especially when there are many different teaching strategies. Seminars that involve multidisciplinary problem-based or case-based learning can be introduced during both preclinical and clinical phases to stimulate students’ information assimilation and clinical reasoning in selecting materials used for clinical dental cases [24]. It is possible to think of preclinical case-based learning as a part-whole scaffolding method to help students prepare for clinical teaching and learning [25]. Students may have the chance to understand the “real-world” significance of incorporating the knowledge of materials science in dentistry and combine information related to fundamental materials science and clinical dental disciplines, which would not often happen if the teaching and learning remained separated in discipline-based curricula [19].

Moreover, dental schools may need to consider case-based assessments in lectures, seminars and laboratories, and clinical aspects of an interdisciplinary approach to teaching dental materials science. Teachers from various disciplines should also collaborate to design a single, multi-course assessment that integrates multi-disciplines [24]. Another possible future practice in the teaching and learning of dental materials science courses is a collaborative approach or shared learning. Undergraduate dental students could learn alongside students studying material science, biomedicine, and engineering, which may facilitate the sharing of knowledge and perspectives regarding the sciences behind these materials. This will also enhance their teamwork and communication skills by working with students from other faculties. Nevertheless, future research should identify the readiness of faculty members to implement an interdisciplinary approach for integrating materials science into dentistry and explore the perspectives of vertical and horizontal integration among dental students in an interdisciplinary-based curriculum.

## 5. Conclusions to Integrate Materials Science

A well-designed dental curriculum that emphasises the integration of materials science knowledge into various dental disciplines is needed for future dental graduates to be competent in delivering high-quality oral healthcare services. One method to achieve this is through the horizontal and vertical integration of dental materials science courses. Moreover, innovative, integrated, and collaborative teaching strategies and assessments are essential for students to understand the fundamental concepts of materials science and engineering while applying that knowledge to preclinical and clinical dental practice. Nonetheless, support and encouragement from the university, faculty, and students are essential to the success of implementing interdisciplinary approaches for integrating materials science and dentistry in contemporary dental education.

## Data Availability

Not applicable.
